# Contemporary clinical perspectives on chronic low back pain: The biology, mechanics, etc. underpinning clinical and radiological evaluation

**DOI:** 10.1002/jsp2.70021

**Published:** 2025-01-23

**Authors:** Stone Sima, Ashish Diwan

**Affiliations:** ^1^ Spine Labs St George and Sutherland Clinical School, University of New South Wales Kogarah New South Wales Australia; ^2^ Spine Service, Department of Orthopaedic Surgery St George and Sutherland Clinical School, University of New South Wales Kogarah New South Wales Australia; ^3^ Spinal Surgery, Discipline of Orthopaedic Surgery, School of Medicine University of Adelaide Adelaide South Australia Australia

**Keywords:** biomechanics, clinical evaluation, discogenic pain, inflammation, low back pain, neuropathic pain, radiology

## Abstract

**Background:**

Pain of a chronic nature remains the foremost concern in tertiary spine clinics, yet its elusive nature and quantification challenges persist. Despite extensive research and education on low back pain (LBP), the realm of diagnostic practices lacks a unified approach. Clinically, LBP exhibits a multifaceted character, encompassing conventional assessments of severity and disability, alongside nuanced attributes like pain characterization, duration, and patient expectations. Common instigators of LBP encountered in spine surgical settings comprise degenerated intervertebral discs (IVD), herniated IVD, canal and foraminal stenosis, and spondylolisthesis. However, addressing the root cause necessitates its identification and substantiation through visualization.

**Methods:**

This perspective reviews the diagnostic complexities of LBP. Thorough history‐taking and physical examinations offer preliminary insights into the underlying source of pain, whether it arises from discogenic origins, neural compression, or sagittal imbalance. The importance of classifying chronic LBP into the underlying pathophysiology is explored. Emphasis is placed on the necessity of aligning clinical observations with imaging findings to guide personalized treatment strategies.

**Results:**

Currently, there exists a disparity globally between evidence‐based recommendations and actual applications. Recent discoveries behind the pathophysiology of pain phenotypes signify the importance of classifying LBP into its neuropathic or nociceptive origins. The pivotal role of radiological investigations in validating clinical findings for an accurate diagnosis cannot be overstated. However, radiology should not operate in isolation; the disconnect between the clinical and radiological realms ultimately benefits neither the patient nor the surgeon. Additionally, more sensitive measures of IVD prolapse and the corresponding inflammatory pathway triggered are required to provide information on the underlying pathophysiological mechanism of pain generation.

**Conclusion:**

This perspective article underscores the imperative fusion of clinical acumen and radiological precision in the intricate landscape of LBP diagnosis. These findings advocate for a paradigm shift towards personalized medicine. By offering a compass for surgeons to navigate this complex terrain and deliver more effective, patient‐centered care with targeted interventions this article aims to enhance management outcomes for chronic LBP.

## Graphical abstract

Pain of the chronic nature remains the foremost concern in tertiary spine clinics, yet its elusive nature and quantification challenges persist. Clinically, low back pain (LBP) exhibits a multifaceted character, encompassing conventional assessments of severity and disability, alongside nuanced attributes like pain characterization, duration, and patient expectations. Radiology should not operate in isolation; the disconnect between the clinical and radiological realms ultimately benefits neither patient nor surgeon. Additionally, more sensitive measures of intervertebral discs (IVD) prolapse, and the corresponding inflammatory pathway triggered is required to provide information on the underlying pathophysiological mechanism of pain generation.

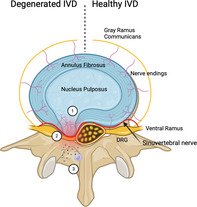

## 1 INTRODUCTION

In plain English, the Oxford Dictionary defines pain as a “highly unpleasant physical sensation caused by illness or injury,” “mental suffering,” or “distress”. Clinically, the International Association for the Study of Pain defines pain as an “unpleasant sensory and emotional experience associated with actual or potential tissue damage.” Ultimately, pain is the opinion of the patient, it is unseen and challenging to quantify.

Low back pain (LBP) is defined by the World Health Organization as “pain inferior to the lowest costal margin and superior to the inferior gluteal folds with or without referred leg pain”.^1^ It stands as a pervasive global public health issue being the leading cause of disability worldwide, affecting over 500 million individuals at any given time and significantly limiting their ability to perform daily activities.^2^ This condition also imposes substantial medical and economic burdens within Australia with LBP representing the second leading cause of total disease burden, accounting for 4.5% of disability‐adjusted life years (DALYs) and costing the government $4.8 billion annually for its management.^3^, ^4^ The point prevalence of LBP is estimated at 14%–25% with a lifetime prevalence rate ranging from 70% to 85%. Beyond its physical impact, LBP is associated with heightened rates of depression, anxiety, and sleep disorders.^5^

Thorough history‐taking and physical examinations offer preliminary insights into the underlying source of pain, whether it arises from discogenic origins, neural compression, or sagittal imbalance. The role of radiological investigations in validating clinical findings for an accurate diagnosis is clinically important. However, radiology should not operate in isolation; the disconnect between the clinical and radiological realms ultimately benefits neither the patient nor the surgeon. Immediate imaging for acute and subacute symptoms often results in misdiagnosis, leading to excessive medical and surgical interventions, which are sometimes unsuccessful in alleviating the patient's symptoms.^6^

However, many diagnostic challenges exist. The most prominent being identifying severe pain in individuals with structurally healthy intervertebral discs (IVDs) and discerning asymptomatic individuals with degenerated IVDs. Literature suggests inflammation and innervation are critical in distinguishing symptomatic from asymptomatic IVDs, yet the precise inflammatory biomarkers initiating pain remain debated. Further novel imaging techniques and investigations are needed to elucidate the underlying pathophysiology of a degenerated IVD.

## 2 UNDERSTANDING LOW BACK PAIN PROFILES

LBP encompasses three distinct patterns of pain: axial lumbosacral, radicular and radiculopathy, and referred pain. Patients suffering LBP are generally classified based on the duration, cause, characteristic, and episodic nature of their symptoms (Table 1).

**TABLE 1:** Common classifications of low back pain.

Low back pain profile	Description
Pattern of pain	
Axial	Pain inferior to the lowest costal margin and superior to the inferior gluteal folds.
Radicular and Radiculopathy	Pain radiating to an extremity in a dermatomal distribution secondary to spinal nerve or dorsal root ganglion irritation. Nerve root irritation leading to neurological deficits such as paraesthesia, weakness, and loss of deep tendon reflexes.
Referred	Low back pain referred to the groin, buttock, and upper thigh. The pain does not have a pattern, is migratory, and rarely radiates below the knee.
Classification of pain	
Chronicity	Acute duration less than six weeks Subacute duration between 6 weeks and 3 months Chronic duration longer than 3 months.^7^
Specific LBP	Presence of symptoms caused by a definitive pathology (i.e. vertebral fracture, malignancy, spinal infection, axial spondylarthritis, ankylosing spondylitis, cauda equina syndrome, etc.).^8^
Nonspecific LBP	Low back pain not attributable to a known specific pathology. Encompassing a wide range of degenerative and idiopathic conditions.
Characteristic of pain	
Nociceptive LBP	Pain due to the activation of peripheral receptive terminals of primary afferent nociceptors that innervate ligaments, small joints, muscle tendons, and other structures in response to noxious mechanical, chemical, or thermal stimuli.^9^, ^10^, ^11^
Neuropathic LBP	Pain caused by a lesion or disease of the somatosensory nervous system.^12^
Episodic nature of pain	
Flare up	Worsening of your condition that lasts from hours to weeks that is difficult to tolerate and generally impacts your usual activities and/or emotions.^13^
Recurrent pain	Return of LBP lasting at least 24 h with a minimum 2 point increase in the numerical rating scale following a period of at least 30 day pain free.^14^

### 2.1 Relevance of leg pain

Radiuclar pain and radiculopathy are often caused by compression or inflammation of the dorsal root ganglia or spinal nerve root.^15^ Both radicular pain and radiculopathy may occur separately or together.^16^ Due to convergence at second‐order neurons, pain may be referred to the low back area. As a result, approximately two‐thirds of patients who present with LBP as their primary concern also present with leg pain. Referred LBP varies widely regarding severity and character, however, is most commonly achy, dull, migratory, and of fluctuating intensity. Referred LBP is unrelated to nerve root compression or inflammation, occurs less frequently than radicular pain or radiculopathy and can be distinguished based on the non‐dermatomal distribution of pain.^17^

Lumbar spinal stenosis, defined as the narrowing of the central or lateral recesses of the canal, can also present with neuroclaudication. Neuroclaudication presents clinically with pain, weakness, and paraesthesia in the lower limbs, exacerbated by activities such as prolonged standing, walking, and lumbar hyperextension. These activities reduce the interlaminar space, which worsens stenosis, leading to direct mechanical or indirect vascular compression of the nerve roots. This group of symptoms often leads to a distinct simian stance posture observed in patients presenting to a spine clinic.^18^ Pain relief often occurs with sitting or forward lumbar flexion.

Vascular claudication shares similarities with neurogenic claudication, presenting as muscle pain in the lower limbs during mild exertion. However, it stems from arterial narrowing, restricting blood flow to the lower extremities. Clinically, it differs from neurogenic claudication in several aspects: pain relief occurs when standing, and posture does not influence symptomatology.^19^ While history and physical examination provide initial diagnostic guidance, additional investigations are necessary to distinguish between the two conditions. Magnetic resonance imaging (MRI) and computed tomography (CT) scans are valuable for revealing mechanical compression of neuronal structures, while doppler ultrasound aids in assessing arterial blood flow.^20^ However, further investigation into the underlying pathophysiology is warranted when patients exhibit claudication symptoms without clear arterial involvement or neuronal compression.

### 2.2 Classification of low back pain

Depending on the specific pathology (i.e. vertebral fracture, malignancy, spinal infection, axial spondylarthritis, ankylosing spondylitis, and cauda equina syndrome), specific LBP can cause compression, and resultant inflammation within the spinal canal, lumbar foramina, or lateral recess leading to NeP.^21^ These conditions may also affect nociceptors present in the IVD, ligaments, paraspinal muscles, and facet joints leading to nociceptive pain (NoP).^22^

Nevertheless, approximately 90% of patients experience not specific LBP, which is characterized by symptoms lacking a clear pathoanatomical cause, rendering it a diagnosis of exclusion. The primary manifestations of not specific LBP are pain and resulting disability.^23^ Not specific LBP is frequently associated with not specific muscular pain and spasms, often attributed to improper lifting, testing, stretching, and other movements.^24^ Notably, imaging studies frequently reveal lumbar spine pathologies such as spondylolisthesis, spondylosis, disc herniation, disc degeneration, and deformity in not specific LBP cases. However, precisely attributing the pain to any of these findings remains diagnostically challenging.^21^ Consequently, these pathologies are more commonly associated with not specific LBP rather than specific LBP, highlighting the intricacies of accurate diagnosis in a clinical context.

### 2.3 Neuropathic vs. nociceptive pain

The characteristics of LBP differ for each person and become complex when viewed from a systemic perspective. On an individual level, it is suggested that pain may stem from tissue damage, mechanical compression, or chemical irritation caused by inflammatory biomarkers.^25^ Currently, research postulates LBP as a linear spectrum with purely nociceptive pain (NoP) on one end and neuropathic pain (NeP) on the other.

NoP may be a result of inflammatory response, mechanical compression, or autoimmune response.^9^, ^10^, ^11^ Nociceptors function as essential protective mechanisms against existing or potential tissue damage. External NoP prompts withdrawal reflexes and fosters adaptive behaviors to avoid similar situations in the future. Similarly, internal NoP acts as a crucial indicator of potential underlying pathologies.^26^

Traditionally, NeP was thought to be a result of mechanical compression of radicular nerve tissues in the spinal canal or foramina. However, it has been hypothesized that inflammatory mediators originating from the degenerated disk and bacteria translocated from the gastrointestinal tract may play a role in the pathogenesis of NeP.^9^, ^10^, ^27^, ^28^, ^29^, ^30^ NeP is hypothesized to bear a worse prognosis and respond ineffectively to traditional analgesics when compared to NoP, as NeP is associated with damage to the underlying radicular nerve tissue and chronicity of symptoms.^31^, ^32^

Numerous scales and questionnaires have been developed to help characterize LBP. The PainDETECT questionnaire acknowledges pain as a spectrum ranging from pure nociceptive on one end to pure NeP on the other.^33^, ^34^ Scores ≤12 suggest an unlikely nociceptive dimension (<15%), while scores ≥19 indicate probable NeP (>90%), and scores between 13 and 18 imply a mixed pain scenario. Subsequent validation demonstrated commendable sensitivity, specificity, and positive predictive values over 80% alongside satisfactory internal consistency and robust test–retest reliability.^34^, ^35^

### 2.4 Clinical importance of pain phenotyping

In clinical practice, there exists a disparity globally between evidence‐based recommendations and actual applications. This is characterized by the underutilization of recommended first‐line treatments and conservative management and the disproportionate reliance on interventions such as imaging, opioids, injections, and surgery. However, these practices fail to address the root causes of LBP‐related disability and mitigate the long‐term consequences of LBP.^36^ Simply put, evidence‐based medicine is not consistently integrated into practice resulting in crucial inadequacies in LBP diagnosis and management. This misalignment is notably evident in the International Classification of Diseases codes and the majority of spine research where all patients suffering from LBP are bundled under one singular entity rather than recognizing its multifaceted nature.^37^

Recent advancements in the understanding of LBP pathophysiology underscore the importance of classifying patients into distinct pain phenotypes.^28^, ^31^, ^35^ The LBP phenotyping consortium has emphasized the importance of categorizing pain as either nociceptive or neuropathic, with the ultimate goal of providing personalized management based on pain phenotypes in patients with LBP.^38^ Difference aspects of pain can either independently or work together to influence how pain is perceived. Nociceptive and neuropathic components of pain might occur simultaneously depending on the patient's conditions. Therefore, it is imperative to associate these pain classifications with underlying pathomechanisms.

Traditionally, studies have described the progression of LBP as disk degeneration simulating the nociceptors of the annulus fibrosus (AF) resulting in NoP which progresses to NeP when degeneration leads to herniation causing pressure on the adjacent nervous tissue.^27^ As a result, many authors believe that NeP is exclusively associated with radiological parameters that demonstrate compression of neuronal structures.^39^ However, recent studies have found that in patients without neuronal compression, those that present with NeP had a higher Pfirmann grade and rate of high intensity zones (HIZs).^28^ This suggests that apart from neuronal compression, disk degeneration itself, and the ensuing hyperinflammation, contribute significantly to the pathogenesis of NeP. HIZs have been proposed to be fluid‐filled zones in the AF resulting from inflammatory edema. These areas exhibit upregulated levels of proinflammatory cytokines, which not only exacerbate neuronal damage but also induce nerve remodeling. Consequently, HIZs are viewed as not only symptomatic but also clinically significant diagnostic indicators.^40^

This has prompted exploration into regenerative biologics aimed at reversing intervertebral disc (IVD) degeneration. While previous attempts were hampered by the risk of adverse events in remote tissues and the potential for accelerated IVD degeneration due to neuronal and vascular ingrowth, recent investigations into growth differentiation factors have shown promise. Growth differentiation factor 6 has demonstrated the ability to enhance glycosaminoglycan production via both SMAD‐dependent and independent mechanisms.^41^, ^42^ These findings underscore the potential of biological therapies in mitigating IVD degeneration. Nonetheless, further research, particularly large randomized controlled trials, is required before the widespread implementation of such therapies in clinical practice.

Due to the degenerative cascade of the damaged IVD (neovascularization, neuronal infiltration, and inflammation) and the alteration of normal force transfer through the spinal column, surgical intervention is targeted at removing the IVD and restoring structure and biomechanical integrity. Many surgical options for interbody fusion of the lumbar spine exist to stabilize the painful motion segment, provide indirect decompression, and restore stability; however, not all are indicated or provide adequate relief from discogenic pain resulting from degenerative disk disease (DDD).^43^ Anterior lumbar interbody fusion approach allows for the complete removal of the degenerated IVD, which has been shown to lead to early pain resolution and high fusion rates.^44^, ^45^

## 3 CLINICIANS VIEW OF LOW BACK PAIN

### 3.1 Pain due to disk degeneration

Degenerate IVD also known as DDD is the most common diagnosis in tertiary spine centers and has the highest prevalence when presenting for spinal surgery. The most common clinical presentation of DDD is LBP with or without radiculopathy.^46^ The pathophysiological process of IVD degeneration begins with changes to the AF. When repetitive trauma and excessive mechanical stresses are exerted onto the lumbar spine the pressure within the IVD significantly increases. A critical point is reached when the AF cannot counteract the forces applied by the NP resulting in AF tears.^47^, ^48^ A disruption in the AF subsequently results in the neuronal proliferation and activation of mechanoreceptors and nociceptors of the sinuvertebral nerve resulting in nociceptive discogenic pain.^49^

**FIGURE 1:**
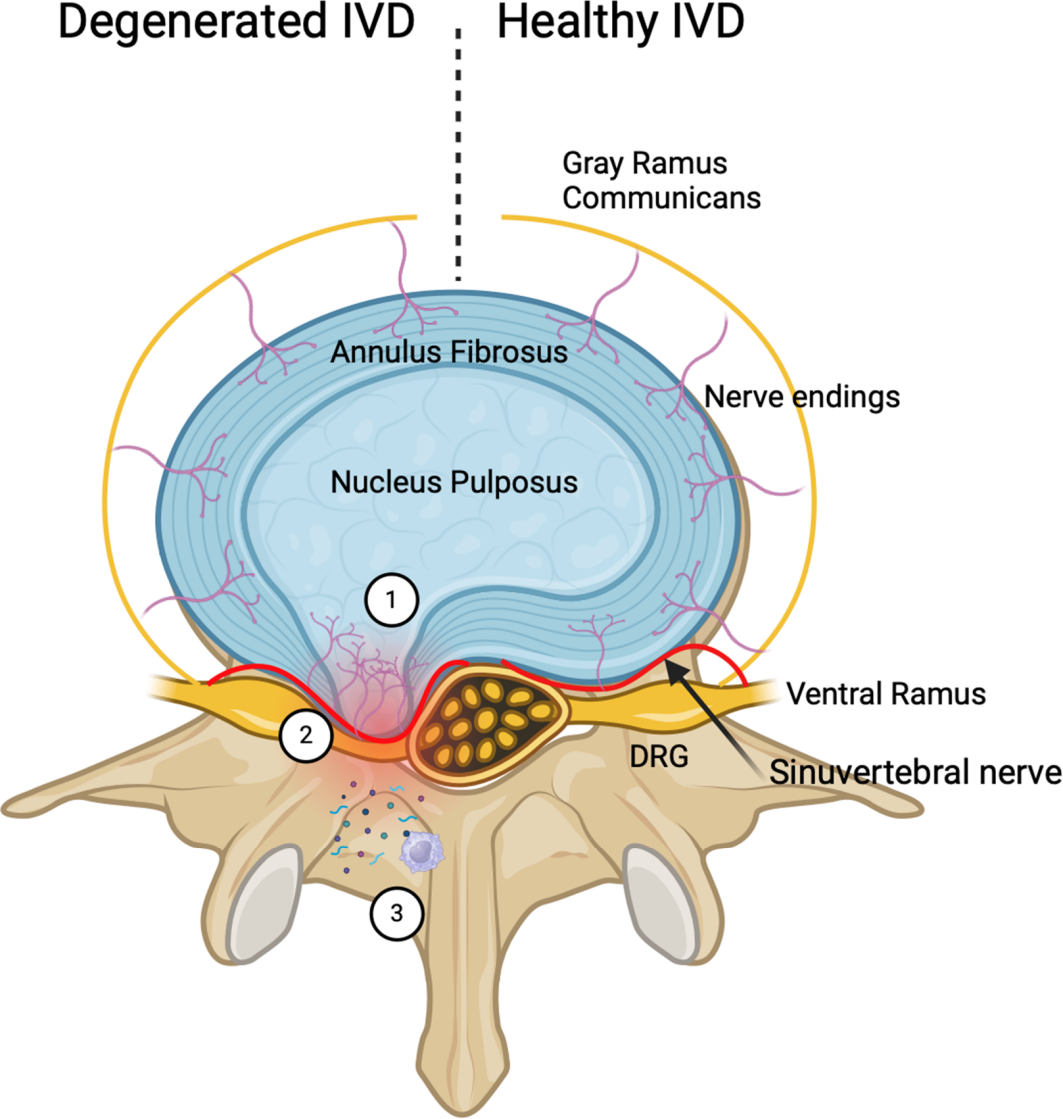
Contrasts a detached nucleus pulposus or contained disk herniation in a degenerated IVD compared to a healthy IVD. In the degenerated disk, there are many possible mechanisms of pain generation. (1) Damaged AF results in proliferation and activation of mechanoreceptors and nociceptors of the sinuvertebral nerve in the AF and nucleus pulposus. (2) Large, herniated disks can lead to mechanical compression and ischemia of surrounding neuronal structures. (3) Proinflammatory biomarkers like macrophages, TNF‐a, C‐reactive protein, and IL‐1 are released as a response to the degenerative IVD and nerve damage.

The mechanism of nervous system response to the degenerate IVD is heavily debated by researchers. Traditionally, a degenerated IVD was only thought to alter the mechanics of the spine resulting in discogenic NoP; however, recent studies posit that the degenerated disk possesses a hyperinflammatory environment that can also result in NeP.^27^, ^28^ Recent studies have linked the disruption of normal IVD structure to the upregulation of neurotrophic factors like calcitonin gene‐related peptide (responsible for neuroproliferation and neuronal remodeling) and proinflammatory markers like tumor necrosis factor‐alpha (TNF‐a), interleukin 1, and prostaglandin E2.^29^, ^50^, ^51^ Both contribute to increased pain perception and neuronal damage that ultimately aids in the pathogenesis of NeP. In severely degenerated disks, the elevated intradiscal pressure can cause the AF to rupture resulting in lumbar disk herniation or extrusion. Mechanically, a herniated disk plus narrowing of the spinal and foraminal canals due to degenerative changes lead to compression or ischemia of the lumbosacral nerve roots.^52^ Subsequently, these morphological alterations may clinically manifest as NeP. However, it is crucial to recognize that acute mechanical nerve compression alone may not precipitate NeP; instead, it commonly results in radicular pain which often resolves post nerve root decompression.^53^ NeP involves the activation of numerous inflammatory and signaling pathways, hypothesized to be stimulated in response to nerve damage. For instance, TNF‐alpha is upregulated in endoneurial macrophages and Schwann cells after neuronal injury, contributing to the manifestation of NeP.^54^ The possible mechanisms of pain generation in a degenerated IVD are illustrated in Figure 1.

### 3.2 Other pain structures

Degeneration of the IVD is also associated with tissue changes in other anatomical structures in the spine and alterations in normal spinal biomechanics, which can also generate pain responses (Table 2).

**TABLE 2:** Alternative anatomical structures responsible for low back pain generation.

Anatomical structure	Mechanism of pain generation
Vertebral body	The degenerated IVD alters the biomechanical loading of the spine resulting in mechanoreceptor activation in the intervertebral body.^55^ Inflammation and mechanical factors like NP rupture can cause bone edema, re‐vascularization, fatty infiltration, and subsequent repair to the endplate of the vertebral body.^56^
Facet joint	The synovial capsule and articulating cartilage possess nociceptive nerve endings. Degenerative osteoarthritis changes cartilage and sub‐chondral bone activates these fibers and induce an inflammatory response. Altered function of the facet joints also leads to instability of the spine^27^, ^57^
Paraspinal musculature	Fatty infiltration of the paraspinal muscles is associated with higher severity of LBP, longer duration of symptoms, and greater expression of inflammatory biomarkers.^58^, ^59^ Muscle atrophy also impairs spinal flexibility, movement, and stability.
Ligamentous structures	Reduced elasticity of the ALL and PLL can result in decreased stability. Degeneration of the IVD may result in reduced disk height and inflammation causing the LF to undergo hypertrophy, which may result in spinal stenosis, nerve root compression or nociceptive activation.^60^

### 3.3 Mechanical instability

In a severely degenerated spine, a combination of IVD degeneration, loss of paraspinal musculature, loss of ligamentum flexibility, and facet joint degeneration can disrupt normal spinal alignment, which can cause the anterior, posterior, or lateral translation of a vertebra in relation to the adjacent vertebrae. This pathological phenomenon is known as degenerative spondylolisthesis (DS) and can result in further deformity. The most common cause of pain in patients with DS is facet arthropathy secondary to joint instability.^61^ This pain is often described as focal to the lower back and nociceptive in nature. Additionally, the translation of the vertebrae can result in altered morphology of the spinal canal and neural foramen which can lead to compression of the nerve root and subsequent upregulation of inflammatory cytokines resulting in NeP. Consequently, the patient suffers from reduced quality of life and often requires a lumbar fusion to improve function.^62^

Although the causative factors of DS are extensively studied, the risk factors and causative mechanism of isthmic spondylolisthesis (IS) remain controversial. IS is defined as the anterior translation of one lumbar vertebra relative to the next caudal segment due to a unilateral or bilateral fracture of the pars interarticularis.^63^ IS can be classified based on underlying etiologies: lytic, characterized by fatigue fractures common in younger adults from repetitive stress; pars elongation, caused by microfractures that heal and elongate the pars, both of which occur in individuals involved in repetitive extension activities; and acute fracture, resulting from severe trauma that leads to immediate anterior vertebral translation.^64^ The progression of spondylolytic defect to IS is associated with a greater chance of LBP and/or radiculopathy due to the potential increased occurrence of instability or disc pathology.^65^ Current literature posits that predictors of progression include factors that increase forces across the pars interarticularis and weaken structures important in maintaining stability. However, a recent systematic review found only disk degeneration to have moderately strong evidence with consistent significant associations with the development of IS.^66^

## 4 CLINICAL CORROBORATION

A clinical diagnosis is essential for pinpointing the structure or lesion responsible for pain. The importance of clinical corroboration and radiological concordance is paramount. A simple juxtaposition of radiological feature to any type of pain and labeling a radiological feature as cause of pain is a known clinical fault. These are the basis of poorly targeted therapies hence consequent poor intervention outcomes.

### 4.1 Role of age in pain

The prevalence of LBP steadily increases from a person's teenage years until 60 years of age and then remains ubiquitous among older adults at retirement ages.^67^ The management of older patients with LBP remains challenging as they often present with more chronic and debilitating symptoms. This population group also suffers from undertreatment and over‐reliance on medical imaging due to difficulties in identifying the underlying cause of LBP. Management of this group should not only focus on underlying pathoanatomical changes in the spine that can cause LBP but also associated comorbidities that are associated with chronic LBP.

Age‐related degenerative changes in the lumbar spine include disk degeneration, hypertrophic facet joints and ligamentum flavum, osteophyte formation, epidural fat infiltration, and paraspinal musculature degradation, which can result in pain due to inflammation, compression or neuronal structures (stenosis), and/or instability (degenerative spondylolisthesis).^68^ The asymmetrical degeneration of the lumbar spine can also result in degenerative scoliosis which is reported to be between 6% to 68% in adults over 60 and results in disabling LBP and significantly diminished quality of life.^69^ Similarly, incidences of vertebral fracture, spinal tumors and spinal infection also increase with age.

Increasing evidence has shown that pain is multifaceted, encompassing a range of physical, biomechanical, chemical, and psychosocial factors. Notably, a higher prevalence of comorbid musculoskeletal disorders, cardiovascular disease, depression, and gastrointestinal conditions has been found in older patients with chronic LBP compared to their non‐LBP counterparts.^30^, ^70^ Other considerations, including cognitive decline, loneliness, changes in pain perception, as well as modified pain assessment methodologies, are required for the management of LBP among older adults.

### 4.2 Clinical history and symptoms

#### 4.2.1 Site of pain

Due to the anatomy of the lumbar vertebrae, the affected dermatome depends on both the level and type of the herniation. The lumbar nerve root transverses through the IVD below its origin and exits laterally, a paracentral herniation will affect the traversing nerve root, and lateral herniation will affect the exiting nerve root of the previous vertebrae. For example, a paracentral L3‐L4 herniation causes L4 radiculopathy while a lateral herniation causes L3 radiculopathy. The corresponding sensory deficit to the dermatome affected is shown in Table 3.

**TABLE 3:** The corresponding sensory deficits of the lumbar dermatomes.^46^, ^71^

Dermatome affected	Sensory deficits
L1	Inguinal region and medial area of thigh
L2	Anterior and medial aspect of upper thigh
L3	Anterolateral thigh
L4	Posterolateral thigh. Anterior tibial area. Area over the patella. Medial aspect of the leg.
L5	Lateral aspect of thigh and knee. Anterolateral aspect of the leg. Dorsum of foot. Big toe
S1	Dorsolateral aspect of thigh and leg. Lateral aspect of the foot

Utilizing sensory tests for diagnosing lumbosacral nerve root involvement demonstrates a notably low sensitivity (0.07 (0.01–0.22) to 0.33 (0.06–0.79)) yet moderate to good specificity 0.58 (0.39–0.75) to 1.00 (0.88–1.00). This suggests that while sensory testing may be valuable in confirming nerve root involvement, it also has a role in ruling out alternative diagnosis.^72^, ^73^, ^74^

#### 4.2.2 Characteristics of pain

NoP typically manifests as localized discomfort centered around damaged tissue, commonly described by patients as sensations of aching, throbbing, sharpness, and dullness.^75^ There is often no underlying neuronal involvement, with symptom severity influenced by mechanical and anatomical factors, and exacerbation or relief of symptoms correlates with proportionate movements.^11^ Contrarily, NeP is characterized by a lesion that induces pain, sensory, and motor deficits localized to the neuroanatomical distribution of the affected nerve.^76^ It is often characterized by patients as burning, tingling, electric shock, and numbness.

### 4.3 Physical examination

Observational findings play a crucial role in discerning the sources of pain. Various indicators can provide valuable insights into differentiating pain origins. For instance, the inability to sit comfortably signifies discogenic pain, while difficulty initiating movement may suggest global osteoarthritis. Patients reliant on a walker often indicate stenosis coupled with sagittal imbalance. Additionally, observing patients with bent knees and a forward stoop posture often signifies sagittal imbalance.

Often patients presenting with LBP and radiculopathy also complains of motor deficits including weakness in the distribution of the lumbosacral nerve roots. The disk level responsible for the affected myotome can be determined via diminished muscle power and gait abnormalities (Table 4).

**TABLE 4:** Affected myotomes resultant from damage to the lumbar nerve roots and their corresponding physical examination findings and deep tendon reflexes.^46^, ^71^, ^77^

Disc level affected	Affected muscle	Examination findings	Deep tendon reflexes
L1‐L2	Weakness in the psoas muscle	Decreased ability to rase thigh in hip flexion test.	Diminished supratellar reflex
L2‐L3	Weakness in the iliopsoas muscle.	Decreased ability for hip flexion, knee extension and hip adduction.	Diminished adductor reflex and patellar reflex.
L3‐L4	Weakness in the quadriceps femoris muscle.	Decreased ability for knee extension and hip adduction.	Diminished patellar reflex
L4‐L5	Weakness in the quadriceps femoris muscle, tibialis anterior muscle, extensor hallucis longus muscle	Decreased ability for foot dorsiflexion and first toe dorsiflexion leading to difficulty heel walking.	Diminished posterior tibial reflex.
L5‐S1	Weakness in peroneus longus, brevis, and gastrocnemius muscle	Decreased ability of for foot eversion and foot plantarflexion leading to difficulty toe walking.	Diminished Achilles and lateral hamstring reflex.

Comparable to sensory tests, motor examinations in studies generally exhibited poor to moderate sensitivity (0.13 (0.04–0.31) to 0.61 (0.36–0.83)) and moderate to high specificity (0.68 (0.49–0.83) to 0.97 (0.85–1.00)). Among the reported studies, foot and first toe dorsiflexion demonstrated the highest sensitivity and specificity. Similarly, patella and Achilles' tendon reflexes both revealed poor to moderate sensitivity (0.14 (0.09–0.21)) to (0.67 (0.21–0.94)) and moderate to good specificity (0.60 (0.51–0.69)) to 0.93 (0.87–0.97).^72^, ^73^, ^74^, ^78^ Thus, while further diagnostic techniques are necessary to confirm weakness and corroborate deep tendon reflex findings, these assessments prove valuable in ruling in the diagnosis of nerve root involvement.

#### 4.3.1 Provocative maneuvers

Provocative maneuvers can be used to screen for lumbosacral radiculopathy or facet joint pathologies. The straight leg raise test is positive when the action of raising the patient's leg to 90 degrees while in the supine position generates an increased pain response in the ipsilateral leg with radiation to the motor/sensory area of the affected nerve root. It usually reveals a disorder of the L5 or S1 nerve root. Reproduction of pain upon performing the femoral stretch test suggests upper lumbar nerve root (L2, L3, L4) disorders.^79^ The literature reports a range of sensitivities and specificities for provocative measures, indicating varying diagnostic performance from poor to good. These tests exhibit both sensitivity and specificity, making them valuable tools for both confirming and excluding the diagnosis of lumbosacral radiculopathy.^80^

The Kemp test, also known as the quadrant test, yields a positive result when the range of motion movements of the lumbar spine elicit localized or radiating pain. Specifically, localized pain during flexion suggests discogenic origin, whereas pain during extension indicates facet joint involvement. Additionally, radiating pain during extension suggests nerve root irritation.^81^

#### 4.3.2 Ruling out the sacroiliac joint and hip as differential diagnoses

It should be noted when patients report LBP, gluteal pain, or anterior thigh pain various other causes than lumbar spinal pathology should be considered, most commonly sacroiliac joint (SIJ) dysfunction and hip pathologies. SIJ dysfunction is one of the most common causes of LBP, accounting for 15%–30% of all cases, and is caused by abnormal motion or malalignment of the sacroiliac joint.^82^ Common examination techniques like the Gaenslen test and posterior shear test exerts forces and stresses on the SIJ and if positive, further diagnostic procedures should be conducted to exclude SIJ involvement.^83^ The FABER test demonstrates high sensitivity and specificity in distinguishing between hip pathology and sacroiliac joint (SIJ) involvement. Pain elicited in the buttock during the FABER test suggests SIJ involvement, whereas pain in the groin region suggests hip pathology.^84^

## 5 RADIOLOGICAL CORROBORATION

The most long‐standing, cheapest, and accessible technique for imaging the spine is via conventional radiography. These are often performed in the anteroposterior and lateral view to evaluate degenerative changes to the anatomical structures of the spine, fractures, spondylolysis, and alignment abnormalities. Flexion and extension X‐rays are also used clinically to evaluate spinal instability in patients with suspected spondylolisthesis.^85^ Common findings of lumbar degeneration and instability on X‐ray are outlined in Table 5.

**TABLE 5:** Degenerative and instability findings on Lumbar X‐ray scans.

Parameter	Description	Role in pain
Disk Height Index^86^	Ratio of the sum of anterior and posterior IVD height to the sum of superior and inferior endplate length	Reduced disk height results in buckling of the posterior ligamentous structures which can result in neuronal compression
IVD angle^87^	Angle between the inferior endplate of the superior vertebrae and the superior endplate of the inferior vertebrae.	Increased IVD angle is associated with increased vertebral misalignment, decreased stability, and hyper lordosis of the lumbar spine
Spinopelvic sagittal balance^55^	Global measurements of the lumbar spine including lumbar lordosis, pelvic tilt, sacral slope, and pelvic incidence	Anterior sagittal imbalance, loss of lumbar lordosis, and increased pelvic tilt have all been found to be associated with a degenerative IVD.
Spondylolytic defects^66^, ^88^	Fracture of the pars interarticularis	Reduces the ability of the posterior elements to stabilize the spine leading to spinal segmental hypermobility
Spondylolisthesis^61^, ^62^, ^87^	Anterior or posterior translation of the lumbar vertebrae	Increases load on the facet joints causing arthropathy. Can also result in canal or foraminal stenosis

Magnetic resonance imaging (MRI) remains the gold standard for imaging soft tissues, providing superior visualizations of the neurological structures, IVDs, ligaments, disks, and paraspinal musculature.^89^ However, MRIs are conducted in a supine position which does not allow clinicians to visualize the spine under loading. The difference in MRI sequences allows different pathological processes to be observed. T2W MRI enhances the signal of water, which has been proposed to represent oedema due to inflammatory processes.^9^ T1W MRI enhances the signal of fatty tissue and suppresses the signal of water.^90^ Coupling of T1W and T2W MRI is necessary to produce a more detailed definition of different spinal pathologies which can provide a higher clinical significance.

Common findings in patients with lumbar degeneration are outlined in Table 6.

**TABLE 6:** Degenerative findings on Lumbar MRI scans.

Parameter	Description	Role in pain
Disk Bulge/Extrusion	Displaced IVD beyond the posterior edge of the adjacent vertebral bodies	Disruption of normal IVD structure resulting in neuroinfiltration and inflammation. In severe cases can cause compression of neuronal structures
Pfirmann Grade^28^, ^91^	A 5 point grade to classify disk degeneration where 1 is a normal IVD and 5 is a severely degenerated IVD	More severe degeneration of the IVD is associated with inflammation, increased prevalence of disk bulge and neuronal compression
Spinal Stenosis^92^	A 4 point grade to classify severity of central canal stenosis where 0 is no stenosis and 3 is severe stenosis.	More severe canal stenosis will leads to compression or ischemia of the traversing nerve roots or cauda equina
Foraminal Stenosis^93^	A 4 point grade to classify severity of foraminal stenosis where 0 is no stenosis and 3 is severe stenosis.	More severe foraminal stenosis will leads to compression or ischemia of the exerting nerve roots
Endplate Changes^56^	Hypointense and hyperintense lesions in the bone marrow and vertebral endplates. Commonly classified via Modic changes, based on the system introduced by Modic et al.^94^	Toxic inflammatory response causing bone edema, re‐vascularization, fatty infiltration, and subsequent repair which differ in presentation on MRI
High Intensity Zones^40^	Hyperintense lesion contained within the AF and apart from the NP observed on T2W MRI where the signal intensity is at least 50% of the cerebrospinal fluid.	Indicative of inflammation. Has been demonstrated as a fluid filled zone resulting from inflammatory oedema or mucoid fluids containing fat. Associated with proliferation of inflammatory cytokines.

Skeletal scintigraphy, commonly known as a bone scan, uses technetium‐99 m as a radiotracer combined with a diphosphonate (methylene diphosphonate or hydroxy‐diphosphonate), which localizes to bone in proportion to osteoblastic activity. Increased uptake in the lumbar spine occurs due to multiple pathological processes such as fracture, infection, inflammation, or malignancy.^95^ Both conventional bone and bone CT scans have been shown to have high reliability for identifying disk pathology and facet disease responsible for pain.^96^ Comparison of bone scan results with conventional imaging like X‐ray or MRI is required to determine underlying pathology and formulate appropriate treatment methods.

### 5.1 Role of spinal pelvic alignment and deformity in pain

Degenerative lumbar scoliosis is a condition characterized by the progressive and asymmetric deterioration of the IVDs and facet joints in adults, which results in sagittal misalignment and three‐dimensional deformity of the lumbar vertebral column. In particular, increased sagittal vertical axis (SVA) and pelvic incidence‐lumbar lordosis (PI‐LL) mismatch are associated with adverse patient reported outcomes pre and post‐surgery as well as negative quality of life and increased disability.^97^ Patients with more severe sagittal imbalance are not only predisposed to undergoing surgical intervention but also exhibit a higher prevalence of adjacent segment disease and non‐union. Additionally, they are at increased risk for experiencing complications like junctional kyphosis and pseudoarthrosis.^98^

The body employs a variety of compensatory mechanisms to restore alignment, underscoring the importance of assessing the entire spine for achieving global sagittal alignment. In response to increased SVA and PI‐LL mismatch, compensatory mechanisms such as heightened pelvic retroversion, diminished thoracic kyphosis, increased knee flexion, elevated pelvic tilt, and augmented pelvic shift are mobilized to achieve sagittal balance.^99^ Surgical intervention, typically through fusion procedures, aims to optimize sagittal alignment parameters, thereby reducing PI‐LL mismatch and SVA to attain global sagittal balance.

However, persistent PI‐LL mismatch and overcompensation of pelvic tilt, along with thoracic hypokyphosis, may predispose individuals to complications such as adjacent segment degeneration, proximal junction kyphosis, and pseudoarthrosis. Moreover, these conditions can contribute to further deformity of the entire spine.^100^ Therefore, preoperative planning must encompass a comprehensive assessment of pelvic orientation and thoracic spine alignment, in addition to evaluating PI‐LL and SVA, to mitigate the risk of postoperative complications and ensure optimal surgical outcomes.

Leg pain is a common symptom of degenerative scoliosis. In these patients, pain is hypothesized to originate from severe degeneration and collapse of the IVD which leads to lateral listhesis, central canal, or foraminal stenosis. Short fusion or direct decompression of the effected IVD in patients with specific dermatomal distribution of pain generally provides satisfactory relief without the patients having to undergo long fusions.^101^, ^102^

However, in cases where pain persists postoperatively, alternative etiologies should be considered. Notably, high fat infiltration within the paraspinal muscles can compromise lumbar spine stability, thereby exacerbating sagittal misalignment. Inflammatory dysfunction of these muscles, attributed to fat infiltration, can lead to heightened expression of pro‐inflammatory cytokines such as tumor necrosis factor‐alpha (TNF‐α) and interleukin‐1beta (IL‐1β), which have been linked to more severe LBP.^58^

### 5.2 Patient case studies

Ensuring alignment between a patient's clinical presentation and radiological findings is essential for effective and accurate management. Presented below are four case studies exemplifying the concurrence of clinical and radiological findings, along with the corresponding treatment modalities employed. These cases underscore the significance of integrating both clinical and radiological assessments to optimize patient care.

**FIGURE 2:**
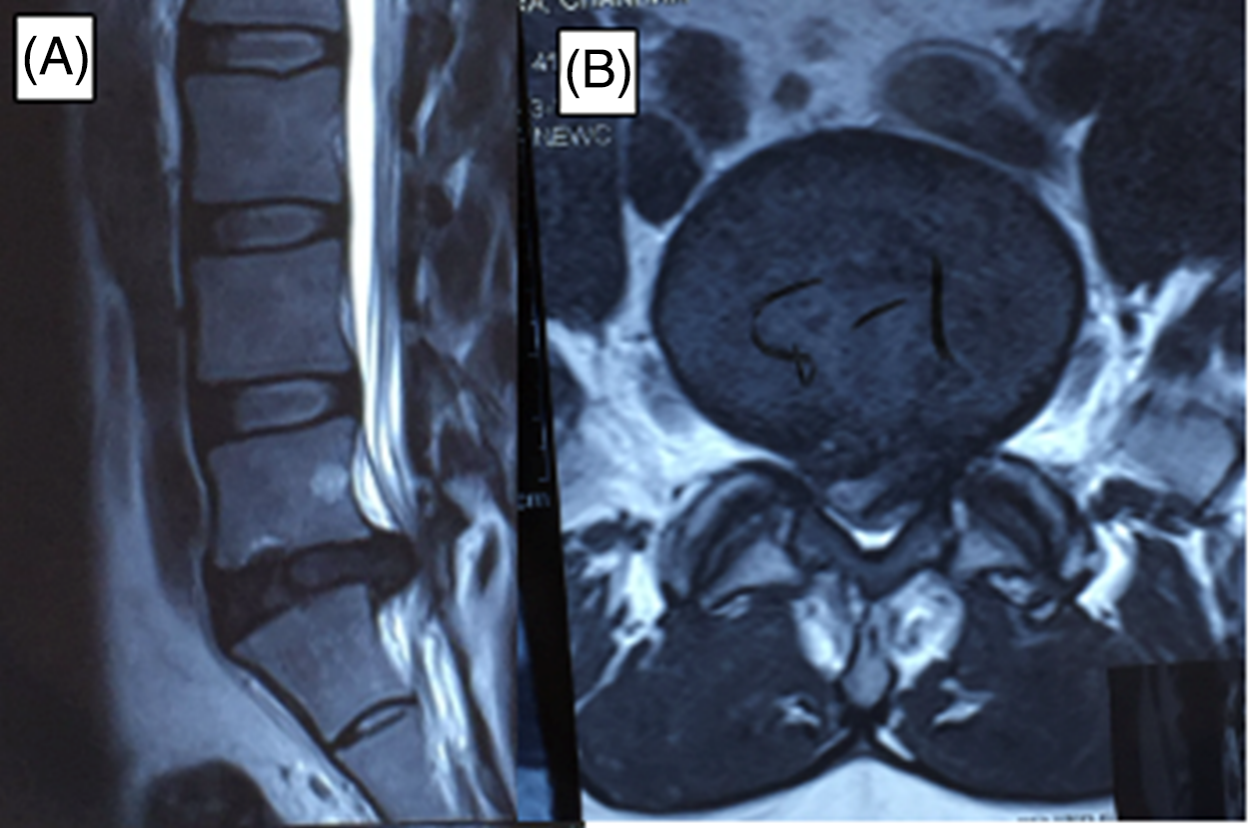
(A) Sagittal MRI showing a large disk herniation at L5‐S1. (B) Axial MRI of the L5‐S1 IVD demonstrates severe canal stenosis (grade 3) resultant from a large central disk herniation which fully compresses the thecal sac.

Case 1 34 years old female who presents with acute bilateral radicular pain (right > left) down the lateral aspect of the lower limbs and foot. The pain was rated as 10/10 using the visual analogue scale (VAS) and the patient reported severe disability with an Oswestry disability index (ODI) score of 60. There was no weakness. Straight leg raise test was positive on the right side only. Deep tendon reflexes were intact and upper motor neuron signs were absent. MRI shows a large central disk herniation of the L5‐S1 IVD. This is causing severe central canal stenosis (grade 3) abutting the traversing S1 and exiting L5 nerve roots (Figure 2). A posterior lumbar decompression and discectomy were performed which alleviated the symptoms. VAS and ODI improved to 2 and 24, respectively, at 12‐month follow‐up.

**FIGURE 3:**
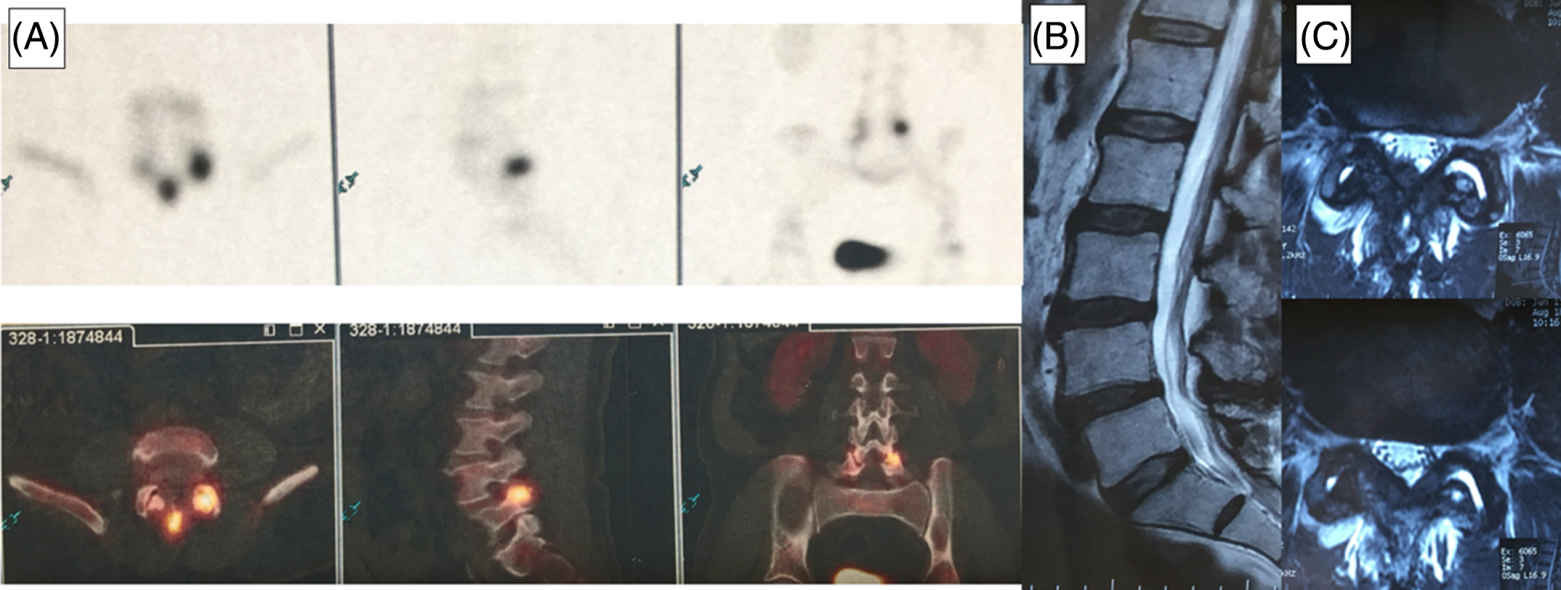
(A) Bone scan demonstrating uptake of Tc‐99 m on the left L4‐L5 facet joint. (B) Sagittal MRI demonstrating no evidence of disk bulge, neuronal compression, high‐intensity zones. (C) Axial MRI of the L4‐L5 level demonstrating a high‐intensity region of the left L4‐L5 facet joint.

Case 2 A 59‐year‐old male who presents with chronic LBP for 6 months. Aggravated by extension of the spine. The pain was rated as 8/10 using the VAS, and the patient reported moderate disability with an ODI of 40. No pain, numbness, or weakness in the lower extremities. Physical examination revealed a positive Kemp test. Deep tendon reflexes were intact and upper motor neuron signs were absent. MRI shows no evidence of disk bulge, neuronal compression, or high intensity zones. However, a high‐intensity region within the left L4‐L5 facet joint was visible, illustrating possible facet joint arthropathy and inflammation. This was confirmed by the bone scan which showed uptake in the left L4‐L5 facet joint (Figure 3). A subsequent facet block of the affected joint was performed which alleviated the symptoms. VAS improved to 2 at post‐injection follow‐up and the patient was subsequently discharged.

**FIGURE 4:**
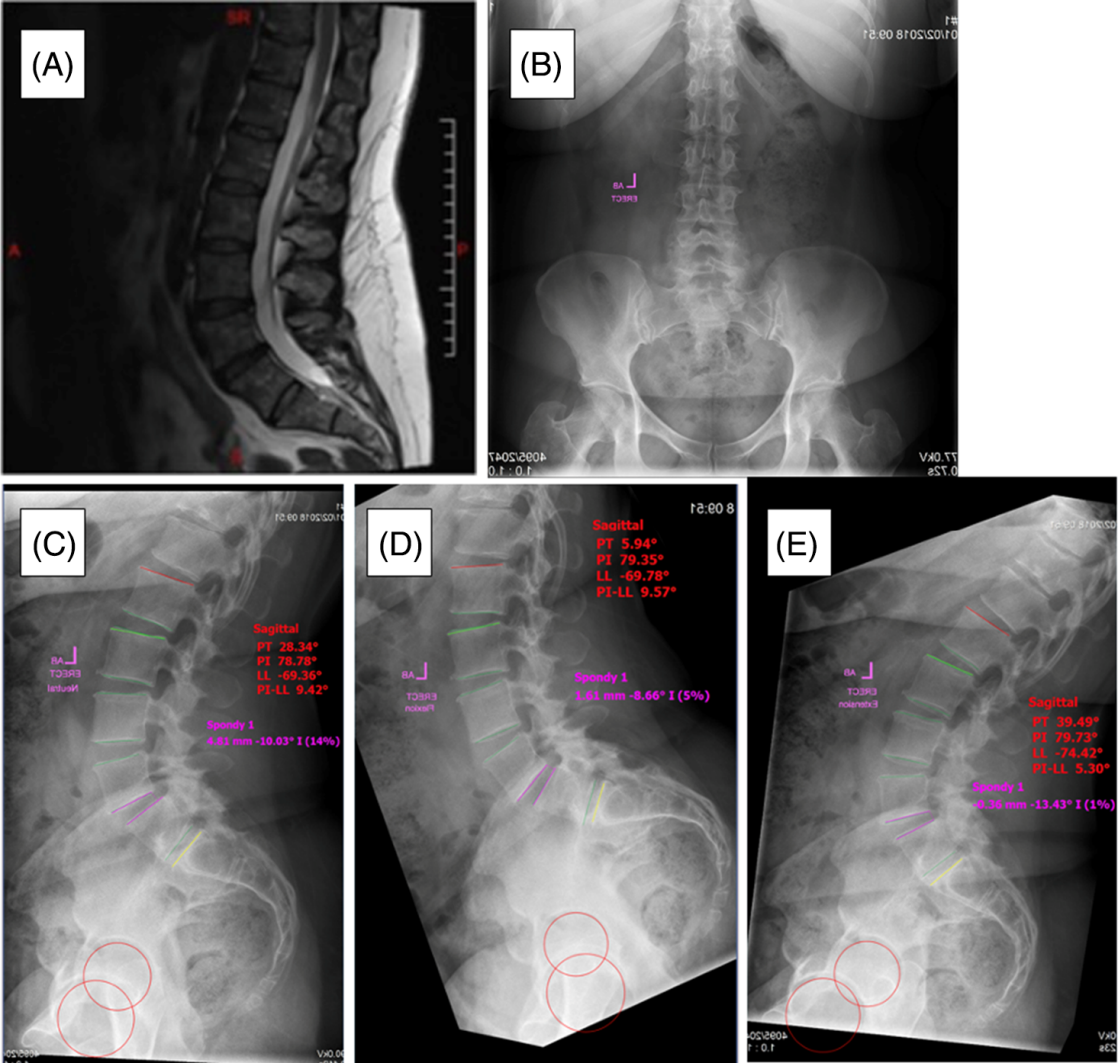
(A) Sagittal MRI demonstrating no evidence of disk bulge, neuronal compression, high‐intensity zones. (B) Anterior X‐ray demonstrating no lateral listhesis or scoliosis deformity. (C) Neutral lateral X‐ray with a 4.81 mm anterolisthesis of L4 on L5. (D, E) Flexion and extension lateral X‐ray demonstrating a reduced anterolisthesis of 1.61 mm and retrolisthesis of 0.36 mm of L4 on L5, respectively. Sagittal alignment parameters were within accepted ranges.

Case 3 A 47‐year‐old female who presents with worsening chronic LBP for 3 years. The pain was rated as 10/10 using the VAS and the patient reported crippling disability with an ODI of 64. No pain, numbness, or weakness in the lower extremities. Physical examination revealed painful restricted motion on flexion of the thoracolumbar spine. Deep tendon reflexes were intact and upper motor neuron signs were absent. MRI shows no evidence of disk bulge, neuronal compression, or high intensity zones. Antero‐posterior X‐ray shows no lateral listhesis or scoliosis deformity. Neutral lateral X‐ray shows normal sagittal alignment with a grade 1 spondylolisthesis (4.81 mm) at the L4‐L5 level. This was reduced to 1.61 and 0.36 mm on the flexion and extension X‐ray, respectively (Figure 4). The patient exhausted all conservative management modalities and a L4‐L5 minimally invasive transforaminal lumbar interbody fusion was performed which alleviated the symptoms. VAS and ODI improved to 4 and 26, respectively, at 12‐month follow‐up.

**FIGURE 5:**
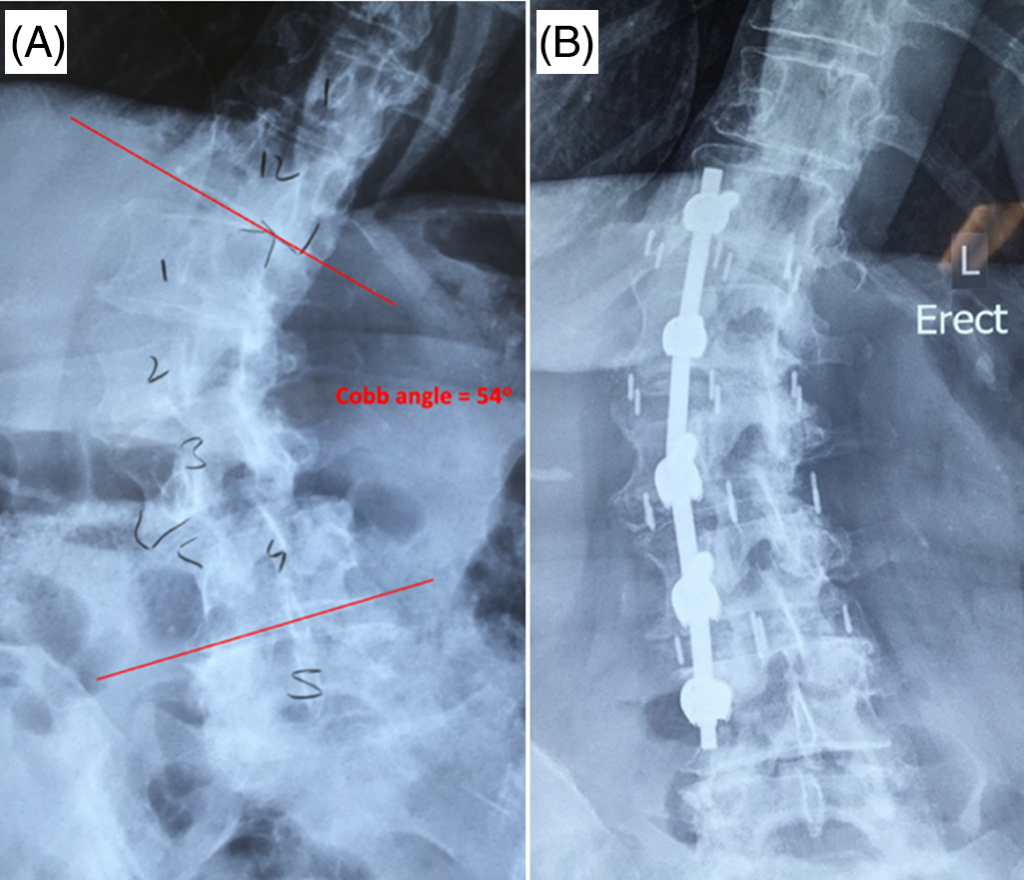
(A) Antero‐posterior X‐ray demonstrating a single right sided lumbar degenerative scoliosis with cobb angle of 54 degrees, multi‐level left sided disk collapse and lateral listhesis. (B) T12 to L4 extreme lateral lumbar interbody fusion with unilateral right sided pedicle screw fixation.

Case 4 A 68‐year‐old female with unremitting chronic LBP, right worst then left, for 6 months. Back pain intensity was rated as 10/10 whilst antero‐medial thigh pain was 5/10. Patient reported crippling disability with an ODI score of 68. Range of movement of the lumbar spine is satisfactory. Deep tendon reflexes were intact and upper motor neuron signs were absent. Antero‐posterior X‐ray shows a single curve right‐sided lumbar degenerative scoliosis with a cobb angle of 54 degrees measured from the T12 inferior endplate to the L4 inferior endplate. Severe left‐sided disk collapse was observed at T12/L1, L1/L2 and L2/L3, with significant lateral listhesis at T12/L1 and L3/L4 (Figure 5). The patient was referred to a pain management clinic and exhausted all conservative treatment modalities including injections. A multi‐level T12 to L4 extreme lateral lumbar interbody fusion with unilateral posterior fixation was performed that alleviated the symptoms. At the 12‐month follow‐up, the patient's VAS scores for back and leg pain improved to 5 and 2, respectively, while the Oswestry Disability Index (ODI) score improved to 30.1.

## 6 DILEMMA OF NO CORROBORATION

The field of spinal research grapples with two predominant diagnostic challenges: identifying individuals with severe pain despite presenting with structurally healthy IVDs and discerning asymptomatic individuals with degenerate IVDs. Existing literature suggests that the persistent presence of inflammation and innervation plays a crucial role in distinguishing symptomatic IVDs from asymptomatic ones.^103^ As previously discussed, degeneration of the IVD is recognized as a precursor to the infiltration of nociceptive nerve fibers into the disc and the escalation of proinflammatory cytokines. However, the precise inflammatory biomarkers responsible for initiating this pain remain a subject of considerable debate. Additionally, in symptomatic patients lacking apparent degenerative findings and neural compression on imaging, there is a need for comprehensive exploration into the underlying catalysts for inflammation and the resulting symptomatic outcomes. Therefore, a more sensitive measure of IVD prolapse that can also provide information on the underlying pathophysiological process is required.

### 6.1 Novel radiological approaches

The current assessment of IVD degeneration relies heavily on qualitative grading systems, such as those proposed by Pfirrmann and Thompson, based on clinical MRI scans.^91^, ^104^ However, the field is rapidly advancing with the introduction of quantitative MRI techniques like T_2_* and T1‐rho relaxometry, which offer insights into degenerative changes at the molecular level.^105^, ^106^ Recent studies have demonstrated the predictive value of T_2_* relaxometry for evaluating sulfated glycosaminoglycans (S‐GAG) content, a key marker of IVD degeneration, highlighting its potential role in assessing disc health and function.^105^, ^107^

Despite promising advancements, challenges persist in implementing quantitative MRI techniques clinically, including technical complexities and time‐intensive postprocessing requirements. Additionally, there is some resistance among radiologists toward quantitative imaging due to concerns about productivity and financial incentives.^108^ Recognizing the need for improved imaging modalities, particularly for animal and preclinical models of disc degeneration, researchers have proposed novel approaches such as multi‐echo MRI sequences.

Recent advancements in postprocessing algorithms for multi‐echo MRI sequences offer a promising avenue for noninvasive and efficient assessment of IVD degeneration. A study by Sheldrick et al. introduced a novel post‐processing method called decay variance, which assigns a score to represent IVD health, demonstrating a stronger correlation with histological grading in a rabbit model compared to other quantitative MRI methods. This technique exhibited high sensitivity and specificity, surpassing 90%, and proved to be more time efficient, underscoring its potential for reliable and rapid evaluation of IVD degeneration.^109^ These innovative approaches hold great promise in deciphering tissue states and enhancing the accuracy of degeneration assessments in clinical and research settings.

### 6.2 The role of inflammation

**FIGURE 6:**
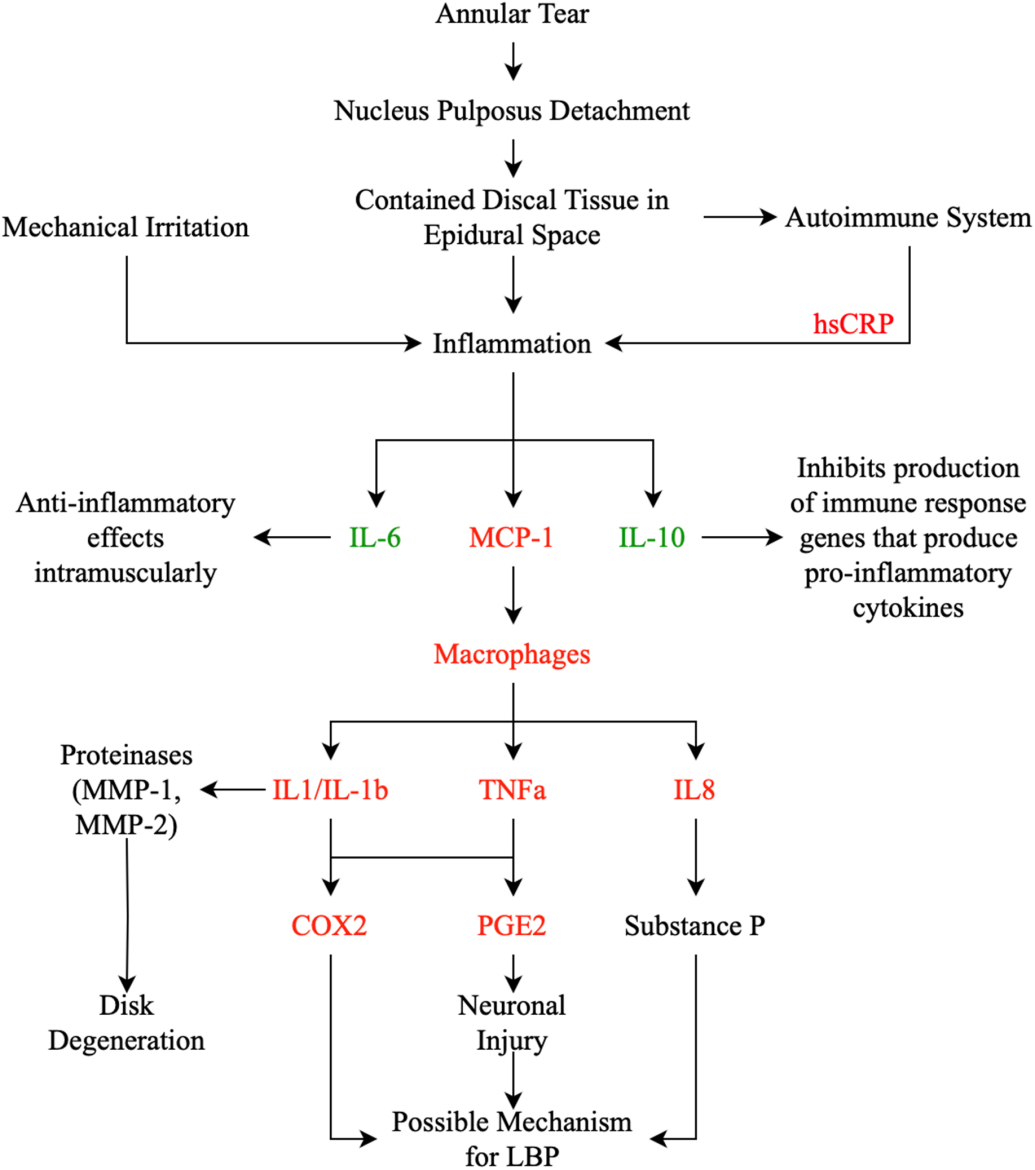
Role of biomarkers in the generation of low back pain. The cytokines colored in red have pro‐inflammatory effects, whilst those colored in green are anti‐inflammatory.

The proliferation of cytokines is initiated by inflammation stemming from the damaged IVD. Notably, a recent systematic review and meta‐analysis revealed a positive correlation between pain severity and disability scores with levels of pro‐inflammatory cytokines. Moreover, a significant reduction in pain and disability post‐treatment is associated with a similar decrease in levels of pro inflammatory and elevation of anti‐inflammatory cytokines.^29^ Mechanical irritation and autoimmune responses also contribute to the activation of inflammatory mediators. Interestingly, the ensuing inflammatory cascade also produces mediators that have anti‐inflammatory and disk resorption activities (Figure 6).^110^

Herniated NP material triggers an auto‐immune response that lead to elevated production of C‐reactive protein (CRP).^111^ CRP is a homopentameric acute‐phase inflammatory protein, traditionally serving as an indirect indicator of systemic inflammation. While the primary role of CRP involves activating the C1q molecule in the complement pathway, it also binds to Fc receptors of IgG, leading to the release of proinflammatory cytokines implicated in LBP.^112^, ^113^

IL‐6 possesses a dual mechanism, acting as a pro‐inflammatory cytokine in monocytes and macrophages through the NFkB pathway, and an anti‐inflammatory myokine when expressed intramuscularly.^114^ Additionally, IL‐10 is an anti‐inflammatory cytokine that inhibits antigen presentation in dendritic cells, macrophage activation, infiltration, and the production of immune‐response genes essential for pro‐inflammatory cytokines.^115^

Monocyte chemoattractant 1 (MCP‐1) promotes chemotaxis of macrophages to the source of MCP‐1.^116^ Macrophages have been agreed to be the most found cells in acute and chronic degenerative IVDs and play an important role in upregulating interleukin 1 (IL‐1), tumor necrosis factor‐alpha (TNFa), and IL‐8.^51^, ^117^

IL‐1 induces upregulation of genes that encode matrix‐degrading enzymes (matrix metalloproteases (MMPs)); hence, it has been found to be present at the site of degenerated IVD and in patients with LBP.^9^, ^118^ Interestingly, MMPs are also effective in degrading cartilage matrix that aids in the degradation of herniated disk material.^119^ Increasing concentrations of IL‐1 also upregulated production of prostaglandin E2 (PGE2) and cyclooxygenase‐2 (COX2), which are thought to be associated with pain hypersensitivity.^9^, ^120^ IL‐8, a chemokine primarily produced by macrophages, plays a pivotal role in inducing chemotaxis of neutrophils and granulocytes, ultimately stimulating phagocytosis. The resulting neurogenic inflammation triggered by neutrophils releases substance P, contributing to pain.^121^ TNFa is a potent proinflammatory cytokine with the capacity to stimulate interleukin production and exert chemoattractant effects. This cascade ultimately upregulates PGE2, a pivotal inflammatory cytokine associated with nerve root injury and the manifestation of a positive Lasegue sign.^122^

A damaged IVD improperly transfers the downward axial load through the NP, leading to mechanical irritation of the posterior AF caused by pivot‐point forces. This results in the posterior shift of axial loads, triggering the nociceptors of the AF.^123^ Blain et al. demonstrated that mechanical stress increases the activity of MMP‐2 and 9 in articular cartilage, illustrating heightened turnover of IVD tissue and resulting in a weakened disc.^124^ Kang et al. also observed an elevated representation of proinflammatory cytokines, specifically MMPs, PGE‐2, and IL‐6 in the discs affected by HIZ.^9^ The confluence of chemical inflammation and physical irritation of the nociceptors creates a detrimental cycle, fostering ongoing inflammation and the generation of LBP.

### 6.3 The role of the bacterial infiltration

Recent research has implicated microbiome‐induced inflammation in the development of disc degeneration and back pain.^125^ A cross‐sectional study conducted in 2019 involving 537 elderly men found a significant association between dysbiosis and the presence and severity of musculoskeletal pain, particularly in the lower back.^126^ Furthermore, Propionibacterium acnes, a commensal skin bacterium known for its involvement in bone and joint infections, has been consistently found in high concentrations within degenerated discs of patients with chronic LBP and neck pain.^127^, ^128^ Expanding on this, significant findings from 2020 by Rajasekaran et al. revealed elevated levels of pathogenic bacteria from the gut in degenerated and herniated disc specimens, whereas healthy discs contained abundant protective bacteria commonly found in the gut.^129^ These discoveries challenge the conventional belief of intervertebral disc sterility and strongly suggest the involvement of the microbiome in the development of LBP.

Overall, these findings give rise to the proposed gut–disk axis as a possible mechanism of IVD degeneration and LBP. A recent study led by Wentian Li et al. suggests potential mechanisms of bacterial infiltration and infection, injury induced inflammation, and release of inflammatory metabolic and cytokine products from the dysbiotic gut to potentiate IVD degeneration and LBP. It hypothesizes that gut microbes alter the intestinal microbial environment by facilitating the translocation of bacteria across the gut epithelial barrier and into the IVD. This not only allows the gut microbiome to regulate the mucosal and systemic immune system but also allows it to regulate nutrient absorption and metabolite formation at the gut epithelium and its diffusion into the IVD.^130^

However, no research yet examines the difference in gut microbiome composition between patients suffering from chronic LBP and asymptomatic controls. Continued exploration into understanding the role of the human microbiome has the potential to significantly enhance patient outcomes through biologics instead of surgery.

## 7 CONCLUSION

LBP is a complex emotional response to underlying injury of associated structures. It can be a response to degeneration, inflammation, instability, or a combination of all. As treating physicians, a comprehensive understanding of the patient's pain presentation, radiological findings, and mechanical considerations is required before formal diagnosis and treatment. As researchers, further understanding of the underlying pathophysiology of pain is required to provide targeted management to match individual patient's needs.
